# Authors’ Reply: Big Data, Small Stories: Methodological Considerations for Using Social Media Analytics in Medical Education Research

**DOI:** 10.2196/97664

**Published:** 2026-06-01

**Authors:** Faisal Binsar, Mohammad Hamsal

**Affiliations:** 1 Binus Online Learning, Management Department, Binus University, Jl. K. H. Syahdan No. 9, Kemanggisan, Palmerah, Jakarta, DKI Jakarta, 11480, Indonesia, 62 215345830 ext 535, 62 215300244; 2 Binus Business School Doctor of Research in Management, Management Department, Binus University, Jakarta, DKI Jakarta, Indonesia

**Keywords:** Icarus paradox, aspirations, realities, medical education, student well-being, community perspective, online reviews

We thank the correspondent for the thoughtful comments [1] regarding our article on the Icarus paradox in Indonesia’s specialist medical education system [2]. We appreciate the opportunity to clarify several methodological aspects of our study, particularly concerning sentiment analysis and the interpretation of neutral discourse in large-scale online data.

Our study was anchored in the Icarus paradox framework, which we applied to the Indonesian specialist medical education system, a context where high aspirations often collide with systemic constraints. The primary objective was to capture the *public pulse* through social listening, a method that provides an unmediated reflection of societal discourse. We maintain that the core findings (identifying the tension between the prestige of specialization and the lived realities of the training system) remain robust. The correspondent’s appreciation of this framework reinforces its utility in analyzing complex health care education phenomena [2].

Brand24 was selected as the primary social-listening tool because it supports large-scale multilingual data collection, including Indonesian-language online discourse. This enabled the analysis of 5047 public responses across digital platforms.

To reduce the limitations of automated sentiment analysis, we implemented manual validation using two independent coders and conducted intercoder agreement checks following established qualitative standards [3]. This process helped ensure that sentiment classification reflected contextual interpretation rather than solely algorithmic outputs.

Although neutral sentiment dominated the dataset, this did not indicate a lack of analytical value. Using NVivo 14 for thematic analysis [3,4], we identified latent structural tensions related to specialist training capacity, workload, and institutional imbalance that aligned with the Icarus paradox framework.

We acknowledge that the use of a single-method sentiment analysis approach is a limitation of the study [5,6]. Future research may benefit from more advanced models capable of distinguishing informational neutrality from subtle positive or negative orientations in online discourse.

We appreciate the correspondent’s methodological reflections and agree that combining automated sentiment analysis with qualitative validation remains essential in digital health research. Our study represents an initial effort to integrate social listening and thematic analysis in examining tensions within specialist medical education in Indonesia. We hope future studies will continue refining these approaches to better understand public discourse in health care education systems.
